# Supplementary material for: Dissociable roles of distinct thalamic circuits in learning reaches to spatial targets
Source: Nat Commun. 2025 Mar 26;16:2962. doi: 10.1038/s41467-025-58143-4 (PMC11947113; doi:10.1038/s41467-025-58143-4)
Supplement: Supplementary file 1 — Supplementary Information [file 41467_2025_58143_MOESM1_ESM.pdf]

Supplementary Figures

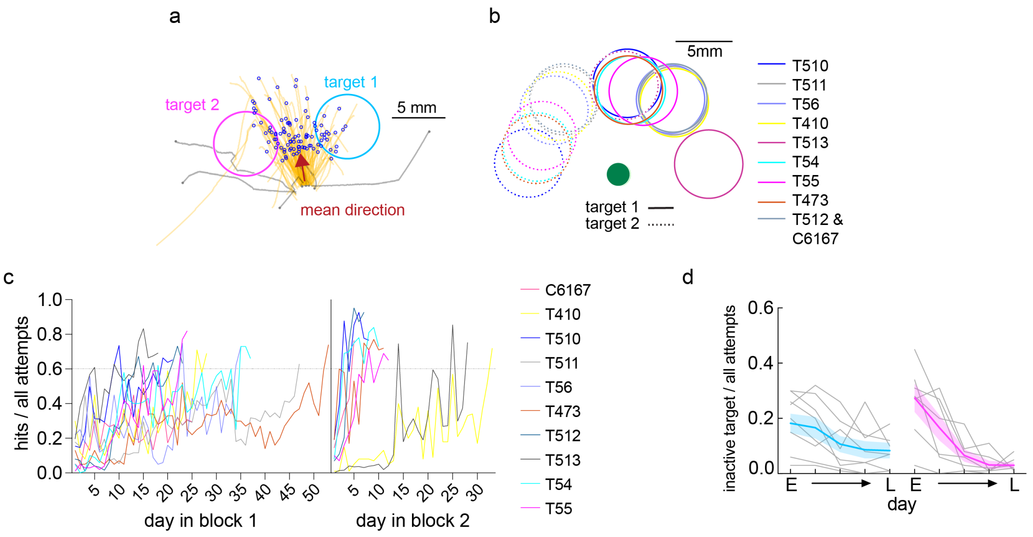

Supplementary Figure 1. Target selection and behavioral training of spatial target task

(a) Representative behavior of a single animal from last day of pre-training. The red arrow shows the mean hit direction from which targets are defined 40° to the left and right.

(b) Individually defined targets for all animals plotted relative to start position (filled green circle). Target 1 for block 1 in solid circle and target 2 for block 2 in dashed circle.

(c) Hit ratio (# hits/ all attempts) for all animals across two blocks of training under 2-photon microscope. Individual animals plotted with separate colored lines.

(d) Inactive target hit ratio. Mixed-effects model, $F(2,17) = 15$, $*p < 0.001$. Mean ± SEM is shown in thick lines with shaded bounds (block 1: blue, block 2: magenta), single animals shown in gray lines (n = 10). Source data are provided as a Source Data file.

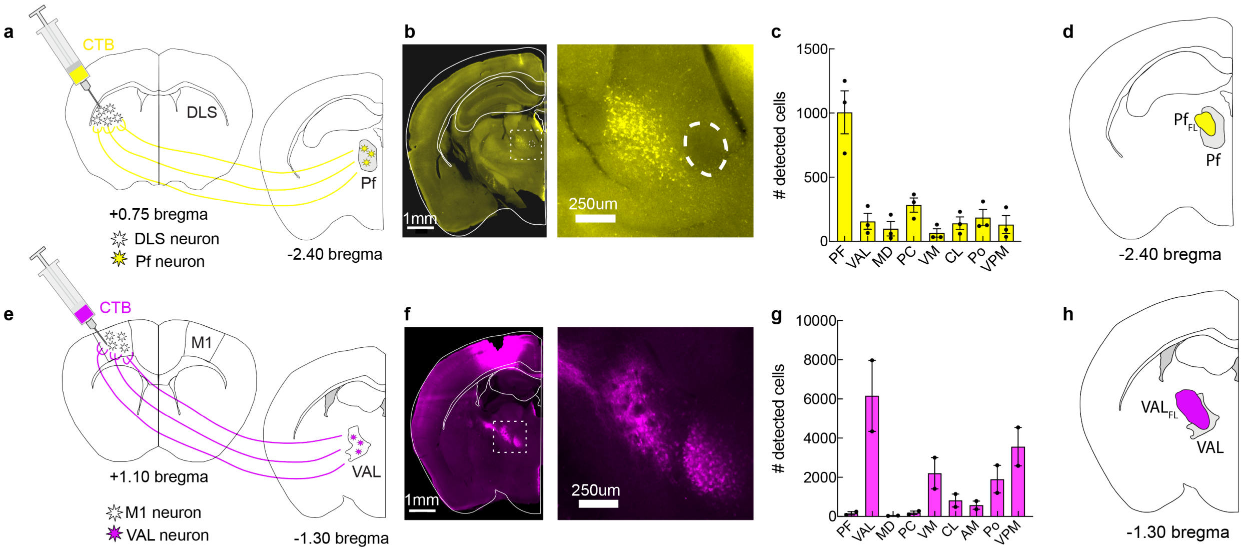

Supplementary Figure 2. Pf and VAL are the dominant forelimb related thalamic nuclei

(a) Schematic of experimental design showing a coronal ABA section at +0.75 mm bregma and CTB injection (yellow) into forelimb DLS and at -2.40 mm bregma showing the center of Pf.

(b) Left: representative section at center of Pf with ABA overlay. Fasciculus retroflexes market with dotted circular outline, and used as an anatomical marker for Pf. Right: magnified image of inset from dashed outline.

(c) Number of detected cells in thalamic nuclei after CTB injection into forelimb DLS (n = 3 mice).

(d) Coronal ABA section at -2.40 bregma highlighting the Pf (gray) with Pf_{FL} defined from CTB tracing overlayed (yellow)

(e) As in (a), but with CTB (magenta) injection in the caudal forelimb area of M1 and ABA sections at the level of M1 and VAL.

(f) Left: representative section at center of VAL with ABA overlay. Right: inset as indicated in dashed outline.

(g) As in (c), but after CTB injection into CFA of M1 (n = 2 mice).

(h) Coronal ABA section at -1.30 bregma highlighting VAL (gray) with VAL_{FL} defined from CTB tracing overlayed (magenta)

(e and f) Mean ± SEM and single animals shown. Acronyms: PF, parafascicular nucleus; VAL, ventroanterior/ventrolateral nuclei; MD, mediodorsal nucleus; PC, paracentral nucleus; VM, ventromedial nucleus; CL, central lateral nucleus; AV, anteroventral nucleus; AM, anteromedial nucleus; Po, posterior complex of the thalamus; VPM, ventral posteromedial nucleus.

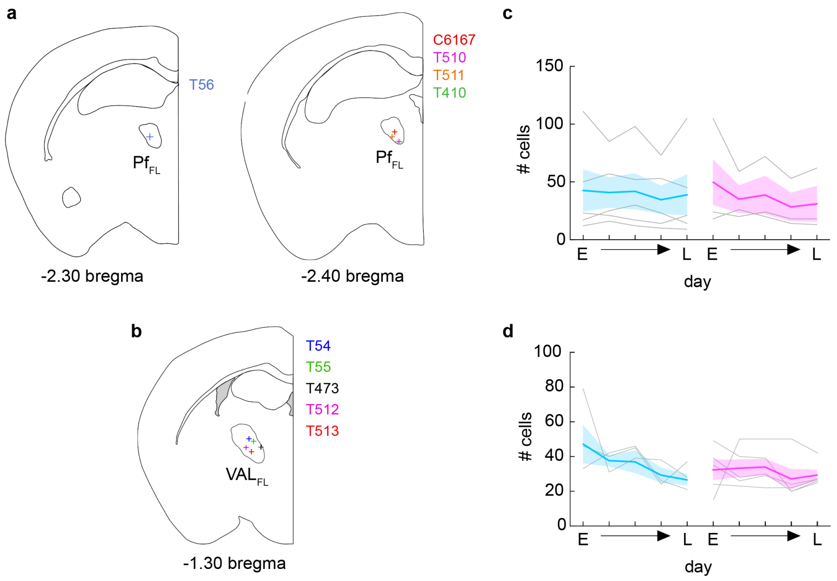

Supplementary Figure 3. GRIN lens locations and number of extracted cells over training of spatial target task

(a) Center of GRIN lens implants for PF imaging animals (+) (n = 5 mice) in coronal plane. Left: coronal plane at -2.30 mm bregma. Right: coronal plane at -2.40 mm bregma. PF_{FL} indicated in outlined section.

(b) Center of GRIN lens implants of VAL imaging animals (+) (n = 5 mice) in coronal plane at -1.30 mm bregma. VAL_{FL} indicated in outlined section.

(c) Number of cells on five days of imaging over block 1(blue) and block 2 (magenta) of STT training. Early (E) day, late (L) day, and 3 equally spaced days over each block.

(d) As in (c), but for VAL animals.

(c-d) Mean ± SEM depicted with thick colored lines and shaded bounds. Individual mice in gray lines.

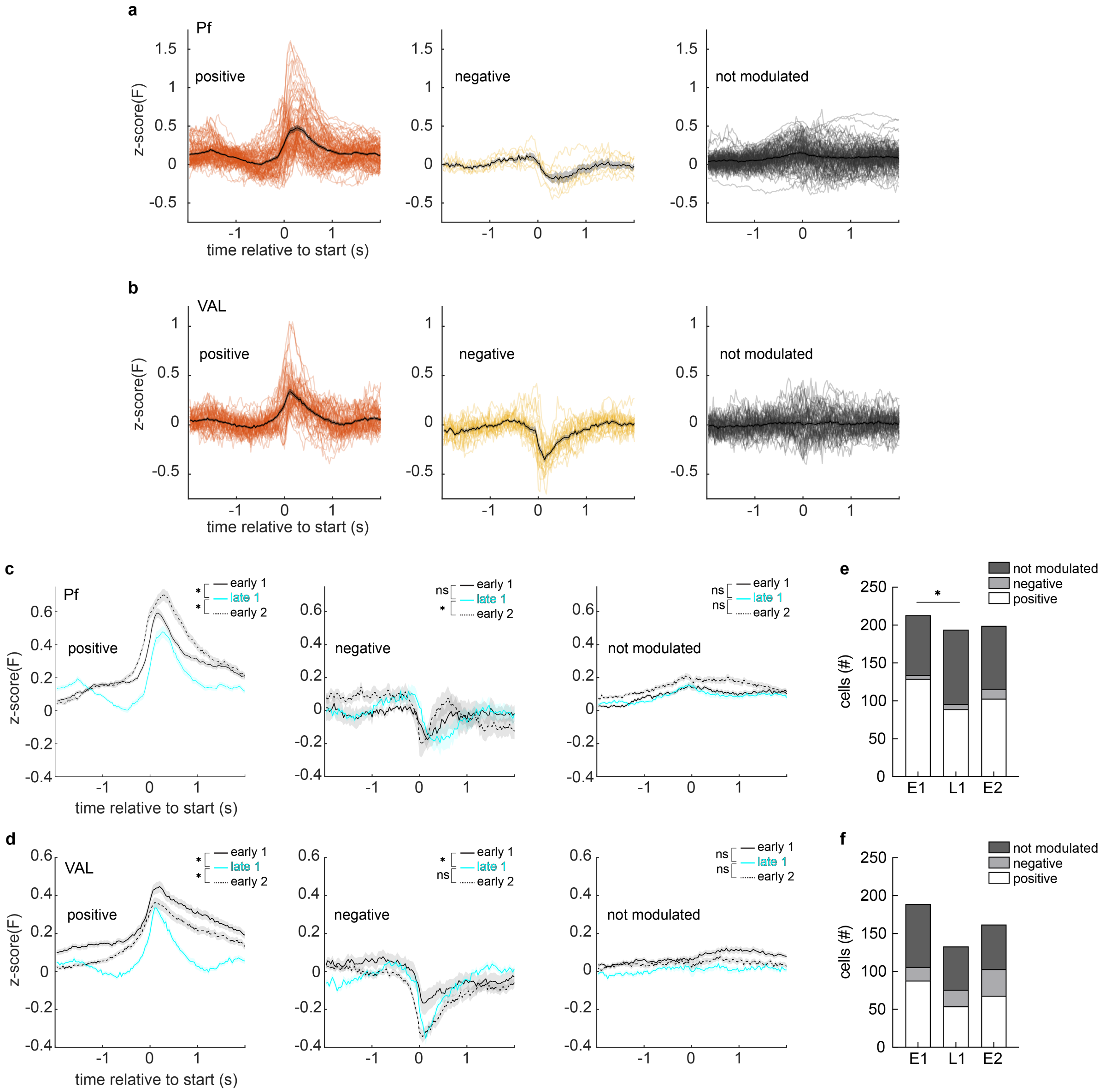

Supplementary Figure 4. Classification of thalamic cells by responses during movement

(a) Representative trial-average responses from positive (orange), negative (yellow), and not significantly modulated (black) cells in Pf from one animal. Mean ± SEM of classified groups plotted in thick black line with shading overlayed.

(b) Same as (a), for VAL.

(c) Change in response for Pf positively (left), negatively (middle), and not modulated (right) cells on early block 1 (solid black line), late block 1 (cyan line), and early block 2 (dashed black line). Kruskal-Wallis test, positive population activity at start: *$p < 0.0001$; Dunn's multiple comparisons test, early block 1 vs late block 1: *$p < 0.0001$, early block 2 vs late block 1: *$p = 0.0001$. Kruskal-Wallis test, negative population activity: *$p < 0.05$; Dunn's multiple comparisons test, early block 1 vs late block 1: *$p < 0.05$, early

block 2 vs late block 1: $p > 0.05$. Kruskal-Wallis test, not modulated population activity: $p > 0.05$; Dunn's multiple comparisons test not significant for all pairs.

(d) As in (c), but for VAL. Kruskal-Wallis test, positive population activity: $*p < 0.0001$; Dunn's multiple comparisons test, early block 1 vs late block 1: $*p < 0.0001$, early block 2 vs late block 1: $*p = 0.0001$. negative population: $*p < 0.01$. Kruskal-Wallis test, negative population activity: $*p < 0.0001$; Dunn's multiple comparisons test, early block 1 vs late block 1: $*p < 0.001$, early block 2 vs late block 1: $p > 0.05$. Kruskal-Wallis test, not modulated population activity: $p > 0.05$; Dunn's multiple comparisons test not significant for all pairs.

(e) Number of Pf_{FL} cells that are positively modulated (white), negatively modulated (light gray), or not modulated (dark gray), during the movement window on the early day of block 1 (E1), late day of block 1. Fisher's exact test: early block 1 vs late block 1: $*p < 0.05$. Late block 1 vs early block 2, $p > 0.05$.

(f) Same as (e), for $VAL_{FL.}$ Fisher's exact test: early block 1 vs late block 1, $p > 0.05$. Late block 1 vs early block 2, $p > 0.05$.

(e and f) Fisher's exact test Pf vs VAL early block 1: $*p < 0.005$, late block 1: $*p < 0.001$, early block 2: $*p < 0.001$

(c and d) Brackets show Kruskal-Wallis test for population activity at start, asterisks depict Dunn's multiple comparisons test $p < 0.05$. Data shown as mean ± SEM (thick colored line with shaded bounds). Source data are provided as a Source Data file.

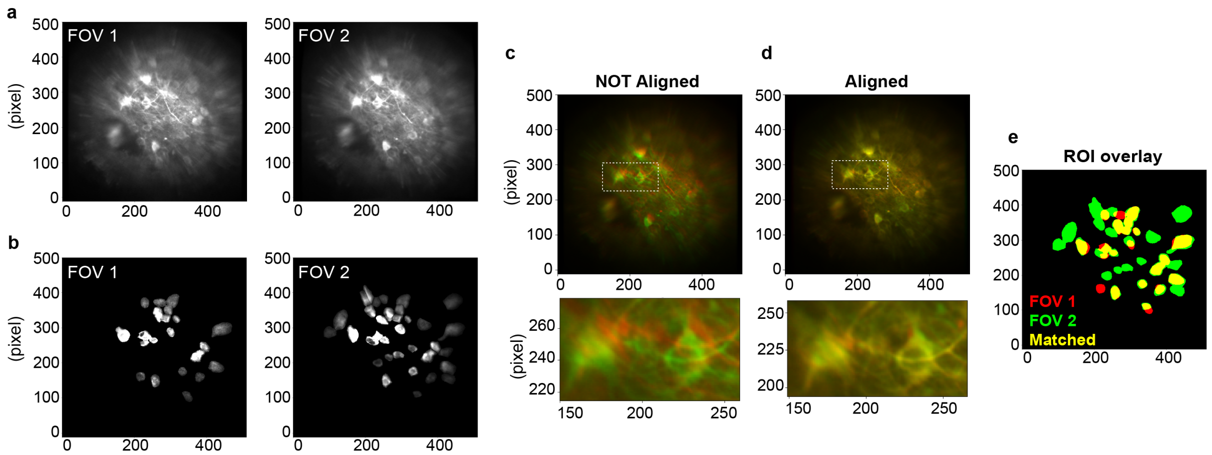

Supplementary Figure 5. Tracking cells over multiple days of 2-photon imaging

(a) Average projections of example imaging FOV over two days. FOV1 from the late day of block 1 and FOV2 from the early day of block 2.

(b) Max projections of detected ROIs from the imaging FOV1 and FOV2 as seen in (a).

(c) Top: Representative overlay of FOV1 and FOV2 before non-rigid alignment; late day of block 1 (red) and early day of block 2 (green). White dotted line indicating inset. Bottom: Inset area before alignment

(d) Top: Representative alignment of FOV1 and FOV2 after non-rigid alignment; late day of block 1 (red) and early day of block 2 (green). White dotted line indicating inset. Bottom: Inset area after alignment.

(e) Overlay of detected ROIs from FOV 1 (red), FOV 2 (green) and matched overlapping areas (yellow) from both sessions.

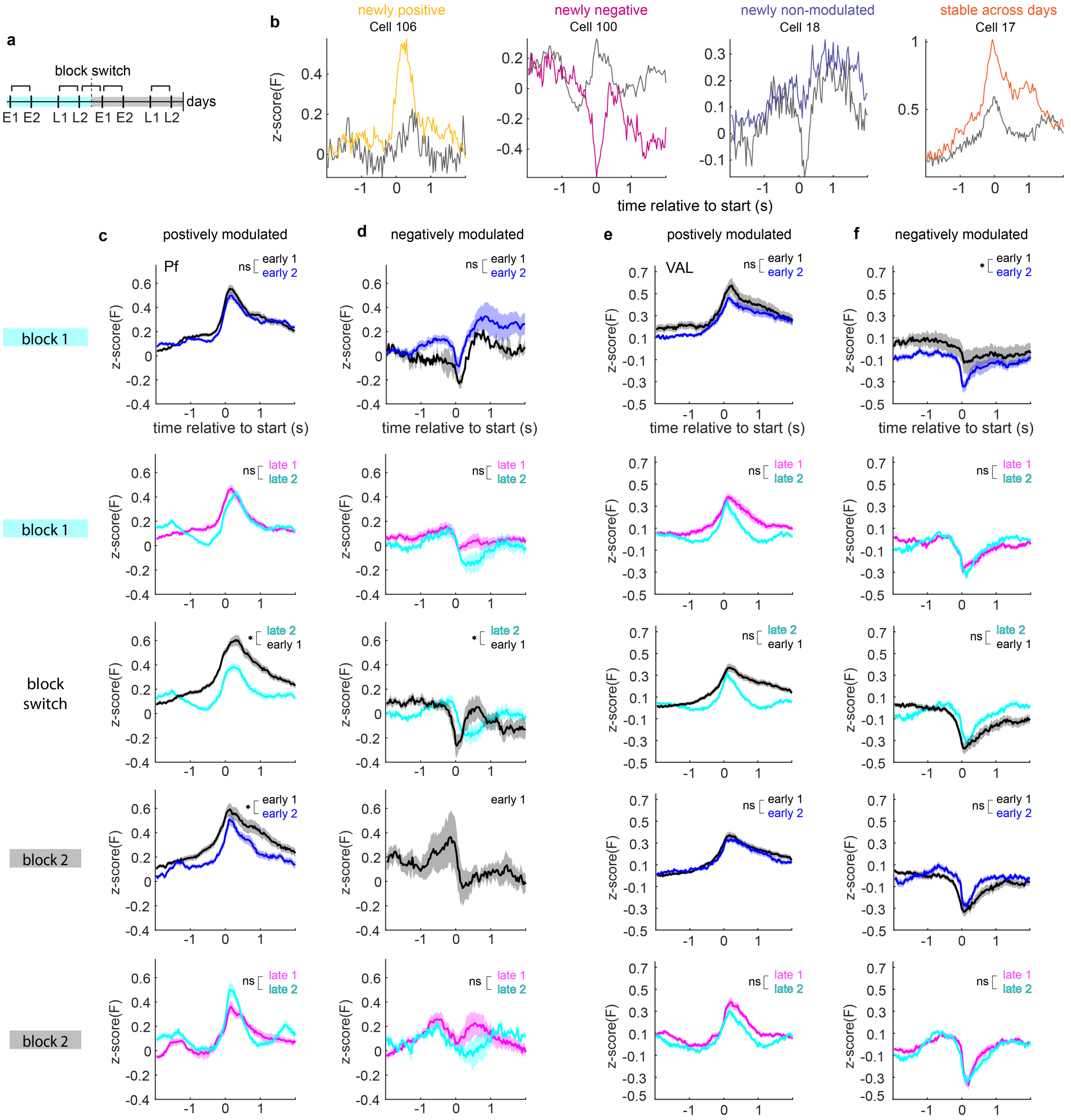

Supplementary Figure 6. Changing matched cellular responses during movement over multiple days

(a) Schematic depicting days for cell matching analysis. Two early days (E1 and E2) and two late days (L1 and L2) for each block were used. The late block 1 day and early block 2 day (L2 and E1) were also used to cell match across the training block switch.

(b) Example trial-averaged traces of matched Pf_{FL} cells during movement on the late day of block 1 (gray line) and early day of block 2 (colored lines).

(c) For Pf_{FL} matched cell populations, trial-averaged fluorescence aligned to movement start for positively modulated cells. Mann-Whitney test, fluorescence at time of movement start: early 1 vs early 2 (block 1) $p > 0.05$, late 1 vs late 2 (block 1) $p > 0.05$,

late 2 (block 1) vs early 1 (block 2) *p < 0.0001, early 1 vs early 2 (block 2) *p 0.05, late 1 vs late 2 (block 2) p > 0.05.
(d) Same as in (c) for negatively modulated cells. Mann-Whitney test, fluorescence at time of movement start: early 1 vs early 2 (block 1) p > 0.05, late 1 vs late 2 (block 1) p > 0.05, late 2 (block 1) vs early 1 (block 2) *p < 0.0.05, early 1 vs early 2 (block 2) p > 0.05, late 1 vs late 2 (block 2) p > 0.05.
(e) For VAL_{FL} matched cell populations, trial-averaged fluorescence aligned to movement start for positively modulated cells. Mann-Whitney test, fluorescence at time of movement start: p > 0.05 for all paired days.
(f) Same as in (e) for negatively modulated cells. Mann-Whitney test, fluorescence at time of movement start: early 1 vs early 2 (block 1) *p < 0.05. For all other paired days, p > 0.05.
(c - f) Data shown as mean ± SEM (thick colored line with shaded bounds).
Source data are provided as a Source Data file.

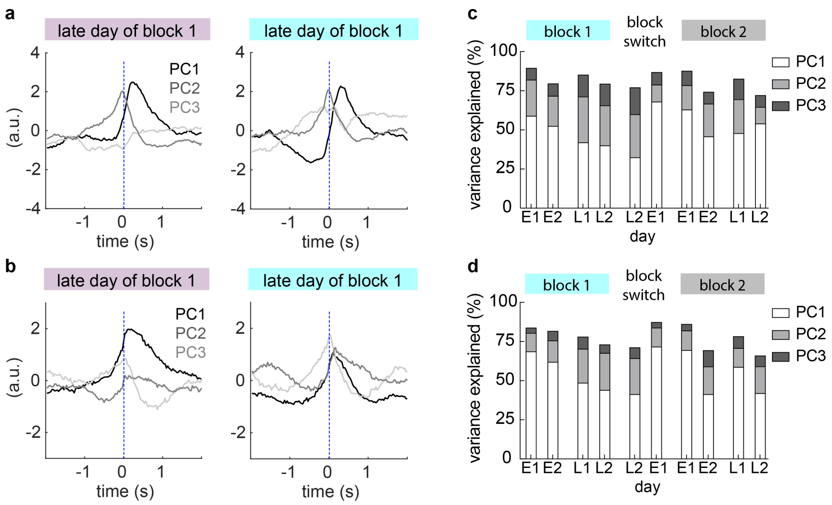

Supplementary Figure 7. Variance explained in low dimensional neural activity of matched cell populations

(a) Top 3 PCs of neural activity of Pf_{FL} around movement for two late days in block 1. Data is plotted against time and aligned to movement start (dashed blue line).

(b) Same as (a) for VAL_{FL} matched neuronal populations.

(c) For Pf_{FL} data, variance explained by PCs 1-3 on pairs of days with cell matching.

(d) Same as (c) for VAL_{FL} matched neuronal populations.

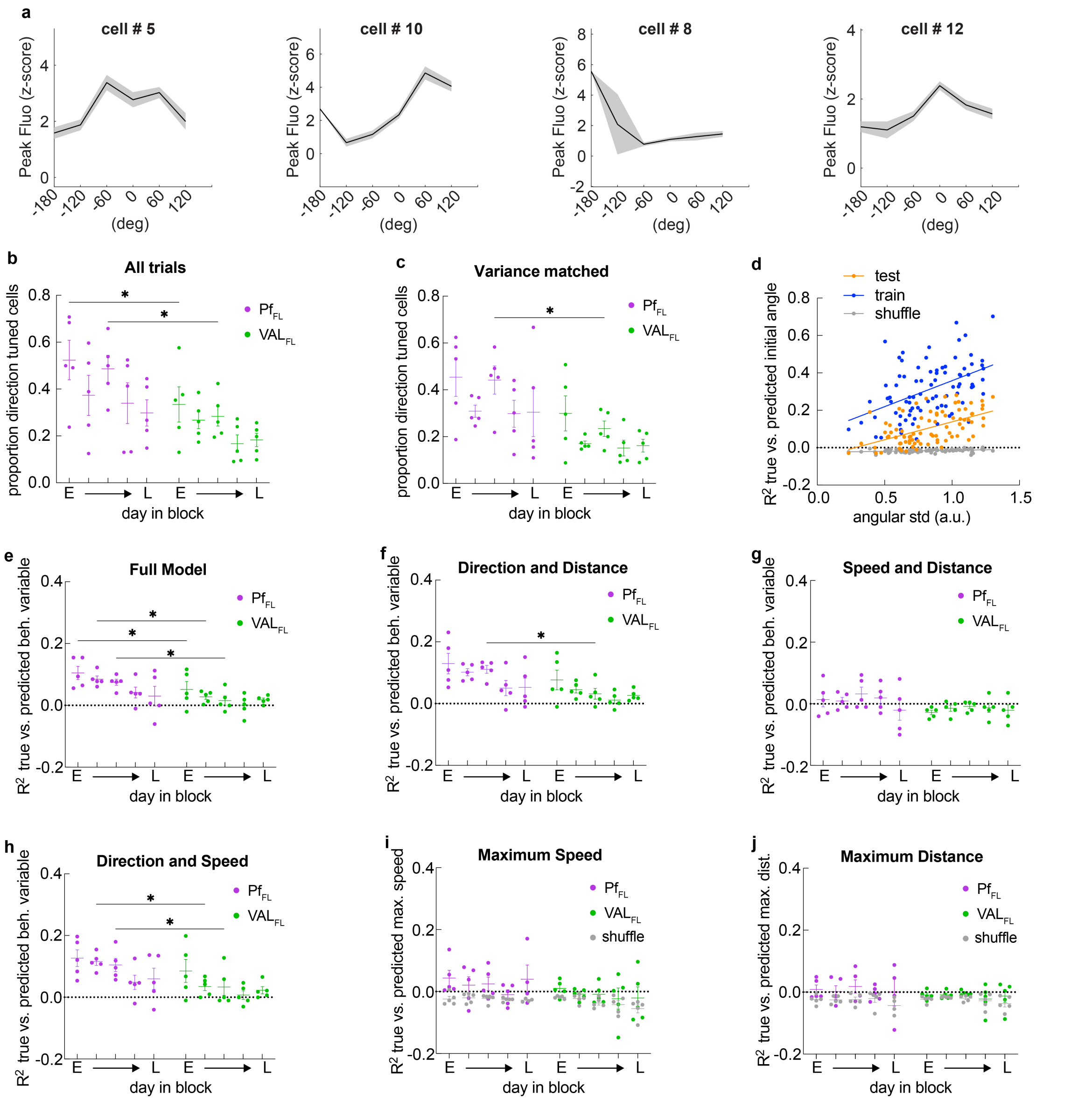

Supplementary Figure 8. Direction tuning and Ridge regressions for reaching variables

(a) Representative examples of cells with directional tuning.

(b) Proportion of direction tuned cells in Pf_{FL} (purple) and VAL_{FL} (green). Data is block averaged for 5 selected days. 2-way repeated measures ANOVA, day effect, $F(4,32) = 4$: *$p < 0.01$, region effect, $F(1,8) = 9$: *$p < 0.05$, Šídák's multiple comparisons test: Pf_{FL} early vs VAL_{FL} early, *$p < 0.05$, Pf_{FL} mid vs VAL_{FL} mid, *$p < 0.05$. For all other day comparisons, $p > 0.05$.

(c) Same as in (b), for trials that are matched for directional variance. 2-way repeated measures ANOVA, day effect, $F(4,32) = 5$: *$p < 0.005$, region effect, $F(1,8) = 7$: *$p < 0.05$, Šídák's multiple comparisons test: Pf_{FL} mid vs VAL_{FL} mid, *$p < 0.05$. For all other day comparisons, $p > 0.05$.

(d) Relationship between the initial vector variability and coefficient of determination (R^2s) from models using all trials on a given day. Linear fit for test (orange), train (blue) and shuffle (gray) models shown. Simple linear regressions, slope different from zero; test (*$p < 0.0001$), train (*$p < 0.0001$), and shuffle (*$p < 0.005$).
(e) Averaged coefficients of determination (R^2s) of full model (direction, distance, and speed) predictions for Pf_{FL} (purple), VAL_{FL} (green) for 5 days of training in each block (averaged over two blocks). 2-way repeated measures ANOVA: day effect, $F(4,32) = 4$: *$p < 0.05$, region effect, $F(1,8) = 14$: *$p < 0.01$. Šídák's multiple comparisons test: Pf_{FL} early vs VAL_{FL} early, *$p < 0.05$. Pf_{FL} early-mid vs VAL_{FL} early-mid, *$p < 0.05$. Pf_{FL} mid vs VAL_{FL} mid, *$p < 0.05$. For all other comparisons, $p > 0.05$.
(f) Same as in (e) for the model predicting trial initial direction and total distance. 2-way repeated measures ANOVA: day effect, $F(4,32) = 4$: *$p < 0.05$, region effect, $F(1,8) = 15$: *$p < 0.01$. Šídák's multiple comparisons test: Pf_{FL} mid vs VAL_{FL} mid, *$p < 0.05$. For all other comparisons, $p > 0.05$.
(g) Same as in (e) for model predicting trial maximum speed and maximum distance. 2-way repeated measures ANOVA: day effect, $F(4,32) = 1$: $p > 0.05$, region effect, $F(1,8) = 3$: $p > 0.05$.
(h) Same as in (e) for model predicting trial initial direction and maximum speed. 2-way repeated measures ANOVA: day effect, $F(4,32) = 3$: *$p < 0.05$, region effect, $F(1,8) = 13$: *$p < 0.01$. Šídák's multiple comparisons test: Pf_{FL} early-mid vs VAL_{FL} early-mid, *$p < 0.05$. Pf_{FL} mid vs VAL_{FL} mid, *$p < 0.05$. For all other comparisons, $p > 0.05$.
(i) Averaged coefficients of determination (R^2s) of maximum speed model predictions for Pf_{FL} (purple), VAL_{FL} (green), and models trained on shuffled vector data (gray). Mixed-effect analysis: day effect, $F(4,31) = 1$: $p > 0.05$, region effect $F(1,8) = 2$, $p > 0.05$. For shuffled data: Mixed-effects analysis, day and region effects, $p > 0.05$.
(j) Same as in (i) for model predicting trial maximum distance. Mixed-effect analysis: day effect, $F(4,31) = 0.5$, $p > 0.05$, region effect $F(1,8) = 1$, $p > 0.05$. For shuffled data: Mixed-effects analysis, day and region effects, $p > 0.05$.
(a-b and e-j) Mean ± SEM is shown for each day, single animals are shown in each point (Pf_{FL}, n = 5 mice; VAL_{FL}, n = 5 mice).
Source data are provided as a Source Data file.

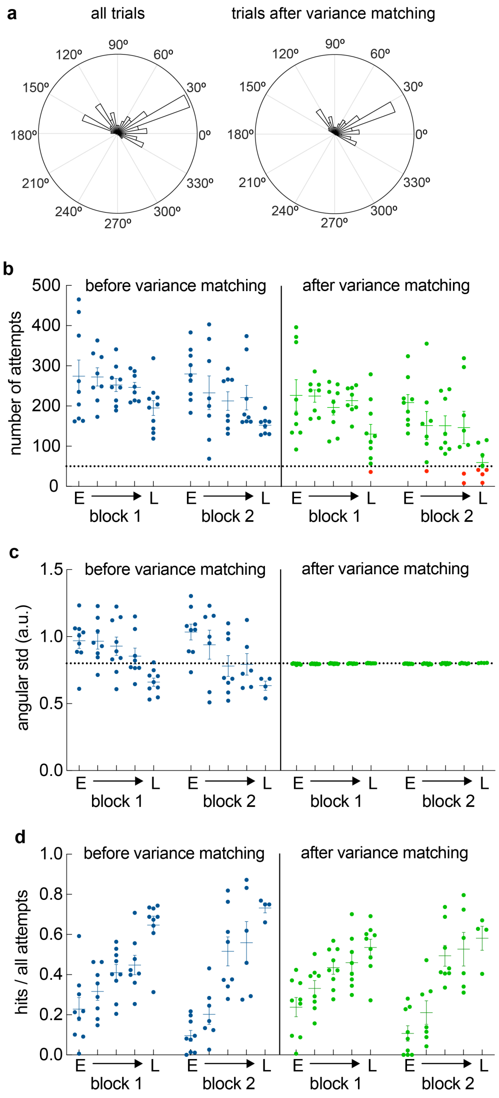

Supplementary Figure 9. Subsampling trials for angular variance matching

(a) Representative polar plot of initial vector direction distribution before (left) and after (right) variability matching.

(b) Number of attempts in 5 training session per block before (left, blue) and after (right, green) variability matching. Red indicates excluded data with less than 50 trials in a session left after variance matching.

(c) Directional variance of initial direction of movement before (left, blue) and after (right, green).

(d) Hit ratio of animals across two training blocks before (left, blue) and after (right, green).

(b-c) Mean ± SEM is shown for each day, single animals are shown in each point (n = 10 mice).

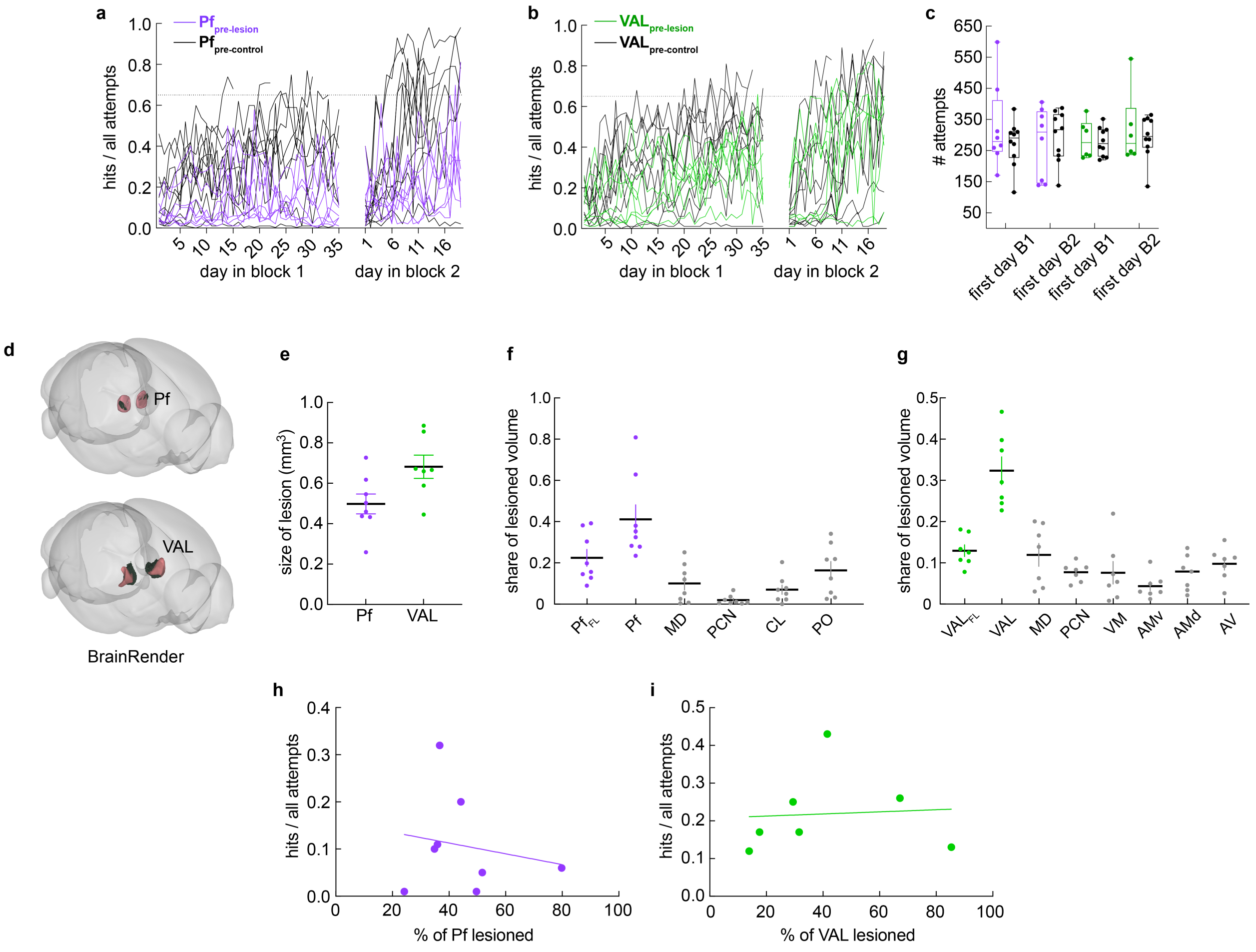

Supplementary Figure 10. Behavioral training of pre-learning Pf and VAL lesioned mice in spatial target task

(a) Hit ratio (hits / all attempts) of each animal in pre-learning lesion experiment. High performance criterion (65% hit ratio) indicated in dotted black line. $Pf_{pre\text{-}lesion}$ (purple), n = 8; $Pf_{pre\text{-}control}$ (black lines), n = 10.

(b) Same as (a) but for VAL pre-learning lesion experiment. $VAL_{pre\text{-}lesion}$ (green lines), n = 7; $Pf_{pre\text{-}control}$ (black lines), n = 10.

(c) Engagement (# of attempts) on the first day of block 1 and block 2. $Pf_{pre\text{-}lesion}$ vs $Pf_{pre\text{-}control}$, unpaired t-test; Block 1: $t(18) = 0.37$, $p > 0.05$, Block 2: $t(18) = 1$, $p > 0.05$. $VAL_{pre\text{-}lesion}$ vs $VAL_{pre\text{-}control}$, unpaired t-test, Block 1: $t(16) = 0.52$, $p > 0.05$, Block 2: $t(16) = 0.13$, $p > 0.05$.

(d) Representative 3D reconstruction of lesions areas (green-volume) of bilateral Pf (top – red volume) and VAL (bottom – red volume) lesion. Made using BrainJ outputs and BrainRender for visualization.

(e) Total lesion volume (mm^3) for $Pf_{pre\text{-}lesion}$ (purple) and $VAL_{pre\text{-}lesion}$ (green) mice.

(f) Relative share of lesioned volume in thalamic nuclei for $Pf_{pre\text{-}lesion}$ mice. Relative share of lesion in forelimb-related Pf and total Pf (purple), other thalamic areas (gray).

(g) Same as in (f) but for $VAL_{pre\text{-}lesion}$ mice. Relative percent of lesion in forelimb-related VAL and total VAL (green), other thalamic areas (gray)
(h) Percent of Pf lesioned in $Pf_{pre\text{-}lesion}$ mice versus hit ratio on the last day of block 1 (hits/all attempts). N = 10 mice. Fitted regression line(purple line) shows there is no significant correlation between percent of Pf_{FL} lesioned and hit ratio. Spearman correlation; Pf_{FL}, r = 0.15, p > 0.05;
(i) Same as (h), but for $VAL_{pre\text{-}lesion}$ lesion cohort, n = 7. Fitted regression line (green) shows there is no significant correlation between percent VAL_{FL} lesioned and hit ratio. Spearman correlation; VAL_{FL}, r = -0.13, p > 0.05. Fitted regression line (green) shows there is no significant correlation between percent VAL_{FL} lesioned and hit ratio.
(e-g) Mean ± SEM shown. $Pf_{pre\text{-}lesion}$ n = 8 mice, $VAL_{pre\text{-}lesion}$ n = 7 mice.
(f and g) Acronyms: MD: mediodorsal nucleus; PCN: paracentral nucleus; CL: central lateral; PO: posterior complex of the thalamus; LP: lateral posterior nucleus; VM: ventral medial nucleus; AV: Anteroventral nucleus; AMv: Anteromedial nucleus, ventral part; AMd: Anteromedial nucleus, dorsal part.
Source data are provided as a Source Data file.

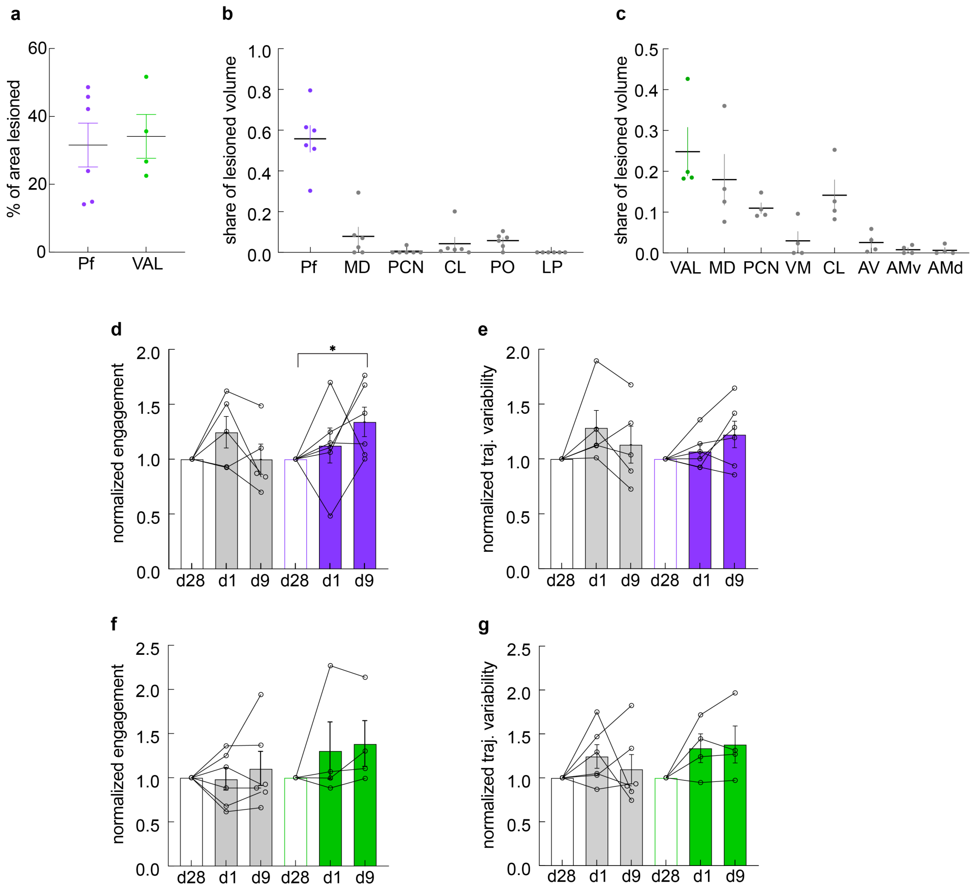

Supplementary Figure 11 Behavioral training of post-learning Pf and VAL lesioned mice in spatial target task

(a) Percent of Pf (purple) and VAL (green) lesioned in post-learning lesion groups.

(b) Relative share of lesioned volume in Pf (purple) and neighboring thalamic nuclei (gray).

(c) Relative share of lesioned volume in VAL (green) and neighboring thalamic nuclei (gray)

(d) Normalized engagement (# of attempts) of Pf groups on d1 and d9 of post-lesion test. $Pf_{post\text{-}lesion}$ group increases number of attempts over 9 post-lesion test days. Two-tailed Wilcoxon test d28 and d9: $Pf_{post\text{-}lesion}$, *$p < 0.05$; $Pf_{post\text{-}control}$, $p > 0.05$.

(e) normalized variability along average trajectory (std in mm) for $Pf_{post\text{-}lesion}$ (purple) and $Pf_{post\text{-}control}$ (gray).

(f) Same as (d), for $VAL_{post\text{-}lesion}$ (green) and $VAL_{post\text{-}control}$ (gray). Two-tailed Wilcoxon test: $p > 0.05$.

(g) Same as (e), for VAL groups. There is no effect for $VAL_{post\text{-}lesion}$ group.

(d-g) Performance metrics on the first (d1) and last (d9) day of post-lesion test. Data normalized to the values on the last day of training before lesion (d28, open white bars). Filled colored bars denote post-lesion days for $Pf_{post\text{-}lesion}$ (purple), $VAL_{post\text{-}lesion}$ (green), and control groups (gray). Individual animal data plotted with open circles. Two-tailed Wilcoxon test, asterisks show Wilcoxon test between d28 and d9.

(a-g) Mean ± SEM and single animals shown.
Source data are provided as a Source Data file.
