# Supplementary material for: Dissociable roles of distinct thalamic circuits in learning reaches to spatial targets
Source: Nat Commun. 2025 Mar 26;16:2962. doi: 10.1038/s41467-025-58143-4 (PMC11947113; doi:10.1038/s41467-025-58143-4)
Supplement: Supplementary file 2 — Reporting Summary [file 41467_2025_58143_MOESM2_ESM.pdf]

nature portfolio

Corresponding author(s): Leslie Sibener, Rui Costa

Last updated by author(s): Feb 24, 2025

Reporting Summary

Nature Portfolio wishes to improve the reproducibility of the work that we publish. This form provides structure for consistency and transparency in reporting. For further information on Nature Portfolio policies, see our Editorial Policies and the Editorial Policy Checklist.

Statistics

For all statistical analyses, confirm that the following items are present in the figure legend, table legend, main text, or Methods section.

n/a	Confirmed	
☐	☒	The exact sample size (*n*) for each experimental group/condition, given as a discrete number and unit of measurement
☐	☒	A statement on whether measurements were taken from distinct samples or whether the same sample was measured repeatedly
☐	☒	The statistical test(s) used AND whether they are one- or two-sided<br>*Only common tests should be described solely by name; describe more complex techniques in the Methods section.*
☐	☒	A description of all covariates tested
☐	☒	A description of any assumptions or corrections, such as tests of normality and adjustment for multiple comparisons
☐	☒	A full description of the statistical parameters including central tendency (e.g. means) or other basic estimates (e.g. regression coefficient) AND variation (e.g. standard deviation) or associated estimates of uncertainty (e.g. confidence intervals)
☐	☒	For null hypothesis testing, the test statistic (e.g. *F*, *t*, *r*) with confidence intervals, effect sizes, degrees of freedom and *P* value noted<br>*Give P values as exact values whenever suitable.*
☒	☐	For Bayesian analysis, information on the choice of priors and Markov chain Monte Carlo settings
☒	☐	For hierarchical and complex designs, identification of the appropriate level for tests and full reporting of outcomes
☒	☐	Estimates of effect sizes (e.g. Cohen's *d*, Pearson's *r*), indicating how they were calculated

Our web collection on statistics for biologists contains articles on many of the points above.

Software and code

Policy information about availability of computer code

Data collection	Software used to collect data included Thorlmage 4, NIS Elements 5.21, TeenScience, Arduino Teensy 3.6, and Bonsai (x64). Custom programs were used in Bonsai, Arduino, and TeenScience to run the spatial target task.
Data analysis	All statistical analyses were performed using Graph Pad Prism 9.5.1/10.1.0. Data processing and analysis were performed in Datajoint, Python 3.7.8, MATLAB 2019b/R2020a, Suite2p 0.10.0, ImageJ 2.1.0. Custom programs in Datajoint, Python, and MATLAB were used to process and analyze data.

For manuscripts utilizing custom algorithms or software that are central to the research but not yet described in published literature, software must be made available to editors and reviewers. We strongly encourage code deposition in a community repository (e.g. GitHub). See the Nature Portfolio guidelines for submitting code & software for further information.

Data

Policy information about availability of data

All manuscripts must include a data availability statement. This statement should provide the following information, where applicable:
- Accession codes, unique identifiers, or web links for publicly available datasets
- A description of any restrictions on data availability
- For clinical datasets or third party data, please ensure that the statement adheres to our policy

The data supporting the findings of this study are available from the authors upon reasonable request.

Research involving human participants, their data, or biological material

Policy information about studies with human participants or human data. See also policy information about sex, gender (identity/presentation), and sexual orientation and race, ethnicity and racism.

Reporting on sex and gender	*Use the terms sex (biological attribute) and gender (shaped by social and cultural circumstances) carefully in order to avoid confusing both terms. Indicate if findings apply to only one sex or gender; describe whether sex and gender were considered in study design; whether sex and/or gender was determined based on self-reporting or assigned and methods used.* *Provide in the source data disaggregated sex and gender data, where this information has been collected, and if consent has been obtained for sharing of individual-level data; provide overall numbers in this Reporting Summary. Please state if this information has not been collected.* *Report sex- and gender-based analyses where performed, justify reasons for lack of sex- and gender-based analysis.*
Reporting on race, ethnicity, or other socially relevant groupings	*Please specify the socially constructed or socially relevant categorization variable(s) used in your manuscript and explain why they were used. Please note that such variables should not be used as proxies for other socially constructed/relevant variables (for example, race or ethnicity should not be used as a proxy for socioeconomic status).* *Provide clear definitions of the relevant terms used, how they were provided (by the participants/respondents, the researchers, or third parties), and the method(s) used to classify people into the different categories (e.g. self-report, census or administrative data, social media data, etc.)* *Please provide details about how you controlled for confounding variables in your analyses.*
Population characteristics	*Describe the covariate-relevant population characteristics of the human research participants (e.g. age, genotypic information, past and current diagnosis and treatment categories). If you filled out the behavioural & social sciences study design questions and have nothing to add here, write "See above."*
Recruitment	*Describe how participants were recruited. Outline any potential self-selection bias or other biases that may be present and how these are likely to impact results.*
Ethics oversight	*Identify the organization(s) that approved the study protocol.*

Note that full information on the approval of the study protocol must also be provided in the manuscript.

Field-specific reporting

Please select the one below that is the best fit for your research. If you are not sure, read the appropriate sections before making your selection.

☒ Life sciences ☐ Behavioural & social sciences ☐ Ecological, evolutionary & environmental sciences

For a reference copy of the document with all sections, see nature.com/documents/nr-reporting-summary-flat.pdf

Life sciences study design

All studies must disclose on these points even when the disclosure is negative.

Sample size	No statistical methods were used to predetermine sample sizes. However, all anatomical observations were repeated using multiple animals, similar to previous studies (Nelson et al., Nature Neuroscience 2021). Behavioral and calcium imaging experiments used sample sizes comparable to previous work (Klaus et al., Neuron 2017; Wagner et al., Cell 2021).
Data exclusions	No data was excluded from this study.
Replication	Attempts to replicate data were successful. Behavioral results were replicated in repeated cohorts of mice with or without various implants, and of multiple mouse genetic strains. Anatomical results were replicated in multiple mice using automated analyses. Calcium imaging and lesion experiments were replicated across mice within multiple experimental cohorts.
Randomization	Randomization was used during analysis in the subsampling of anatomical data, subsampling of trials for training and testing data sets in linear regression analysis, and for shuffling of data for control regression and principal component analysis. Animals within genotype pools were randomly assigned to experimental groups.
Blinding	Blinding was used during all pre and post-training lesion experiments. Experimenters performing surgeries were blinded to the genotype of mice, and were only unblinded after the completion of behavioral training. Blinding was not used for analysis, but automated analyses were used were not subject to experimenter bias.

Reporting for specific materials, systems and methods

We require information from authors about some types of materials, experimental systems and methods used in many studies. Here, indicate whether each material, system or method listed is relevant to your study. If you are not sure if a list item applies to your research, read the appropriate section before selecting a response.

Materials & experimental systems

n/a	Involved in the study
☐	☒ Antibodies
☒	☐ Eukaryotic cell lines
☒	☐ Palaeontology and archaeology
☐	☒ Animals and other organisms
☒	☐ Clinical data
☒	☐ Dual use research of concern
☒	☐ Plants

Methods

n/a	Involved in the study
☒	☐ ChIP-seq
☒	☐ Flow cytometry
☒	☐ MRI-based neuroimaging

Antibodies

Antibodies used: All antibodies used can be found listed in Methods.

Validation: Antibodies were validated through vendor statistics and controls (see Invirtrogen GFP Antibody (A-21311) in western blot).

Animals and other research organisms

Policy information about studies involving animals; ARRIVE guidelines recommended for reporting animal research, and Sex and Gender in Research

Laboratory animals: The strains used were: C57BL6/J (Jackson Laboratories, strain#: 000664), VGlut2-Cre (Jackson Laboratories, strain#: 028863, C57BL6/J background). Prior to surgery, adult mice were housed in groups (2-5 per cage). Following surgery, mice used for behavioral experiments were individually housed with running wheels for enrichment. All mice were kept under a 12-h light-dark cycle.

Wild animals: No wild animals were used in this study.

Reporting on sex: Adult male mice (n=l0), aged 3-6 months, were used for all 2-photon calcium imaging experiments. Adult mice of both sexes (n=26 female, n=32 male), aged 2-6 months, were used for all lesion experiments. Adult mice of both sexes (n=3), aged 4-8 months were used for anatomy experiments.

Field-collected samples: No field-collected samples were used in this study.

Ethics oversight: All experiments and procedures were performed according to National Institutes of Health (NIH) guidelines, with lab protocols approved by the Institutional Animal Care and Use Committee of Columbia University.

Note that full information on the approval of the study protocol must also be provided in the manuscript.

Plants

Seed stocks: *Report on the source of all seed stocks or other plant material used. If applicable, state the seed stock centre and catalogue number. If plant specimens were collected from the field, describe the collection location, date and sampling procedures.*

Novel plant genotypes: *Describe the methods by which all novel plant genotypes were produced. This includes those generated by transgenic approaches, gene editing, chemical/radiation-based mutagenesis and hybridization. For transgenic lines, describe the transformation method, the number of independent lines analyzed and the generation upon which experiments were performed. For gene-edited lines, describe the editor used, the endogenous sequence targeted for editing, the targeting guide RNA sequence (if applicable) and how the editor was applied.*

Authentication: *Describe any authentication procedures for each seed stock used or novel genotype generated. Describe any experiments used to assess the effect of a mutation and, where applicable, how potential secondary effects (e.g. second site T-DNA insertions, mosiacism, off-target gene editing) were examined.*
